# Bis[(*m*-phenyl­enedimethyl­ene)­diammonium] tetra­deca­borate

**DOI:** 10.1107/S1600536808033333

**Published:** 2008-10-18

**Authors:** Xiao Jiang, Shu-Li Wu, Zhi-Dong Shao, Yun-Xiao Liang

**Affiliations:** a State Key Laboratory Base of Novel Functional Materials and Preparation Science, Faculty of Materials Science and Chemical Engineering, Ningbo University, Ningbo, Zhejiang 315211, People’s Republic of China

## Abstract

The title compound 2C_8_H_14_N_2_^2+^·[B_14_O_20_(OH)_6_]^4−^, contains diprotonated C_8_H_14_N_2_^2+^ cations and centrosymmetric tetra­deca­borate anions. The crystal structure is stabilized by O—H⋯O and N—H⋯O hydrogen bonds.

## Related literature

For background on the importance of borate compounds, see: Chen *et al.* (1995 ▶); Grice *et al.* (1999 ▶). For previous work on boron oxoanions, see: Liu *et al.* (2006 ▶); Pan *et al.* (2007 ▶); Grice *et al.* (1999 ▶); Schubert *et al.* (2000 ▶); Touboul *et al.* (2003 ▶); Burns (1995 ▶).
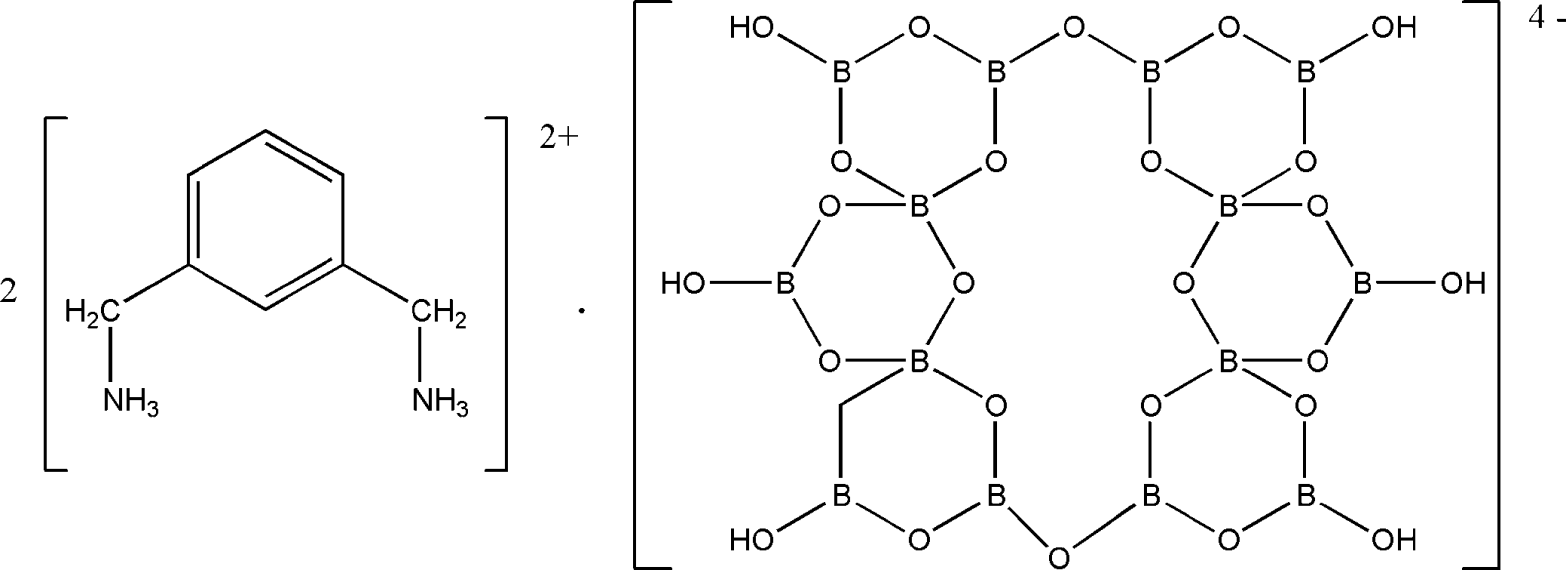


## Experimental

### 

#### Crystal data

2C_8_H_14_N_2_^2+^·B_14_H_6_O_26_^4−^
*M* *_r_* = 849.81
Triclinic, 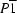

*a* = 9.1025 (18) Å
*b* = 10.293 (2) Å
*c* = 10.942 (2) Å
α = 109.68 (3)°
β = 108.24 (3)°
γ = 102.19 (3)°
*V* = 857.4 (5) Å^3^
*Z* = 1
Mo *K*α radiation
μ = 0.14 mm^−1^
*T* = 295 (2) K
0.34 × 0.26 × 0.18 mm

#### Data collection

Rigaku R-AXIS RAPID diffractometer
Absorption correction: multi-scan (*ABSCOR*; Higashi, 1995 ▶) *T*_min_ = 0.964, *T*_max_ = 0.973
6803 measured reflections
3009 independent reflections
2002 reflections with *I* > 2σ(*I*)
*R*_int_ = 0.023

#### Refinement

*R*[*F* ^2^ > 2σ(*F* ^2^)] = 0.035
*wR*(*F* ^2^) = 0.128
*S* = 1.24
3009 reflections
272 parameters
H-atom parameters constrained
Δρ_max_ = 0.42 e Å^−3^
Δρ_min_ = −0.46 e Å^−3^

### 

Data collection: *RAPID-AUTO* (Rigaku, 1998 ▶); cell refinement: *RAPID-AUTO*; data reduction: *CrystalStructure* (Rigaku/MSC, 2002 ▶); program(s) used to solve structure: *SHELXS97* (Sheldrick, 2008 ▶); program(s) used to refine structure: *SHELXL97* (Sheldrick, 2008 ▶); molecular graphics: *ORTEPII* (Johnson, 1976 ▶); software used to prepare material for publication: *SHELXL97*.

## Supplementary Material

Crystal structure: contains datablocks I, global. DOI: 10.1107/S1600536808033333/bt2801sup1.cif

Structure factors: contains datablocks I. DOI: 10.1107/S1600536808033333/bt2801Isup2.hkl

Additional supplementary materials:  crystallographic information; 3D view; checkCIF report

## Figures and Tables

**Table 1:** Hydrogen-bond geometry (Å, °)

*D*—H⋯*A*	*D*—H	H⋯*A*	*D*⋯*A*	*D*—H⋯*A*
O10—H10*A*⋯O2^i^	0.82	1.98	2.784 (2)	168
O11—H11*A*⋯O3^ii^	0.82	1.84	2.659 (2)	179
O13—H13*A*⋯O11^iii^	0.82	2.00	2.815 (2)	173
N1—H1*A*⋯O7	0.89	2.08	2.863 (2)	146
N1—H1*B*⋯O13^iv^	0.89	1.98	2.850 (2)	166
N1—H1*C*⋯O6^v^	0.89	2.20	2.916 (2)	137
N1—H1*C*⋯O1^v^	0.89	2.54	3.394 (2)	161
N2—H2*A*⋯O4^vi^	0.89	1.97	2.822 (2)	159
N2—H2*B*⋯O1^v^	0.89	1.99	2.877 (2)	173
N2—H2*B*⋯O9^v^	0.89	2.55	3.052 (2)	116
N2—H2*C*⋯O12^vii^	0.89	2.19	3.067 (2)	168

Symmetry codes: (i) 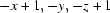; (ii) 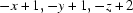; (iii) 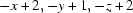; (iv) 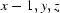; (v) 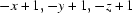; (vi) 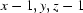; (vii) 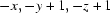.

**Fig. 1.**
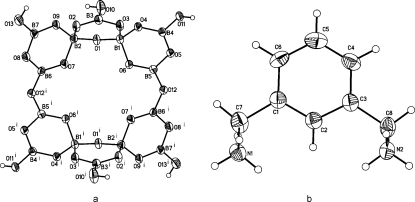
The crystal structure of [(C8H14N2)]2[B14O20(OH)6]: (a) [B14O20(OH)6]4-; (b) [(C8H14N2)]2+, drawn at the 50% probability level. [Symmetry codes: (i) -x+1, -y+1, -z+1.]

**Fig. 2.**
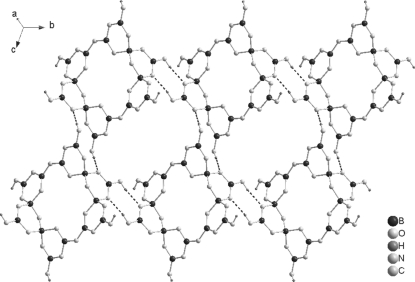
Formation of the two-dimensional sheet from the [B14O20(OH)6]4- polyanions. Hydrogen bonds are indicated by dashed lines.

**Fig. 3.**
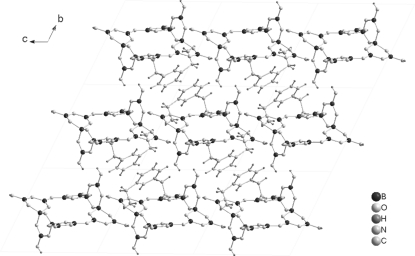
View of the diprotonated of organic amines in the inorganic borate network along a axis.

## supplementary crystallographic information

### Comment

Borate compounds have considerable mineralogical and industrial importance (Chen *et al.*, 1995; Grice *et al.*, 1999). Boron atoms form strong bonds with oxygen atoms not only in trigonal planar BO_3_, but also in tetrahedral BO_4_ groups. These BO_3_ and BO_4_ groups may be linked together by sharing common oxygen to form isolated rings and cages or extended chains, sheets, and networks. So far, a number of isolated boron oxoanions have been found in mineral and synthetic borates, such as [B(OH)_4_]^-^, [B_2_O(OH)_6_]^2-^, [B_3_O_3_(OH)_4_]^-^, [B_4_O_5_(OH)_4_]^2-^, [B_5_O_6_(OH)_4_]^-^, [B_6_O_7_(OH)_6_]^2-^ (Grice *et al.*, 1999; Touboul *et al.*, 2003), [B_7_O_9_(OH)_5_]^2-^ (Liu *et al.*, 2006; Pan *et al.*, 2007), [B_9_O_12_(OH)_6_]^3-^ (Schubert *et al.*, 2000), and [B_14_O_20_(OH)_6_]^4-^ (Liu *et al.*, 2006; Pan *et al.*, 2007). Compared with metal borates, the synthesis of organically modified nonmetal borates was less well explored in the past decades. Herein, we describe the synthesis and crystal structure of a new nonmetal borate with [B_14_O_20_(OH)_6_]^4-^ as polyanions.

As shown in Fig. 1, the title compound consists of isolated [B_14_O_20_(OH)_6_]^4-^ polyborate anions and [C_8_H_14_N_2_]^2+^ cations. The [B_14_O_20_(OH)_6_]^4-^ borate anion is composed of four BO_4_, four BO_3_, and six BO_2_(OH) groups (Burns, 1995). It can also be seen as two [B_7_O_9_(OH)_5_]^2-^ clusters combined with each other through the dehydration of four hydroxyl groups. Each [B_7_O_9_(OH)_5_]^2-^ group contains three B_3_O_3_ cycles held together *via* two common BO_4_ tetrahedra. The B—O bond lengths and O—B—O bond angles are in the range of 1.332 (4)–1.509 (4) Å and 105.9 (3)–124.5 (3)° (Table 1), which are in good agreement with other borates reported previously (Liu *et al.*, 2006; Pan *et al.*, 2007).

In the present instance, the isolated tetradecaborates anions are linked together through hydrogen bonds: O10—H10A···O2, O11—H11A···O3 (Fig. 2), forming a two-dimensional sheetlike structure. The adjacent borate sheets are further linked together by strong H-bonding interactions [O13—H13A···O11] to form a three-dimensional network (Fig. 3). The hydrogen bonds are listed in Table 2.

### Experimental

The title compound was obtained by the reaction of H_3_BO_3_ and 1,3-Bis(aminomethyl)benzene under mild solvothermal conditions. Typically, a mixture of H_3_BO_3_ (0.9882 g), 1,3-Bis(aminomethyl)benzene (3 ml) was stirred at room temperature. The final mixture was sealed in a Teflon-lined autoclave, heated to 443 K at a rate of 10 K/h, kept at 443 K for 4 days and then cooled to room temperature at a rate of 5 K/h. Colorless transparent block-like crystals were collected and dried in air.

### Refinement

All H atoms were positioned geometrically and refined as riding model [O—H = 0.82 Å, N—H = 0.89 Å, C—H_aromatic_ = 0.93 Å, C—H_2_ = 0.97 Å, and with *U*_iso_(H) = 1.5*U*_eq_(O), *U*_iso_(H) = 1.5*U*_eq_(N), *U*_iso_(H) = 1.2*U*_eq_(C)].

### Crystal data

**Table:**

2C_8_H_14_N_2_^2+^·B_14_H_6_O_26_^4^^−^	*Z* = 1
*M**_r_* = 849.81	*F*(000) = 436
Triclinic, *P*1	*D*_x_ = 1.646 Mg m^−^^3^
Hall symbol: -P1	Mo *K*α radiation, λ = 0.71073 Å
*a* = 9.1025 (18) Å	Cell parameters from 4940 reflections
*b* = 10.293 (2) Å	θ = 6.7–54.9°
*c* = 10.942 (2) Å	µ = 0.14 mm^−^^1^
α = 109.68 (3)°	*T* = 295 K
β = 108.24 (3)°	Block, colourless
γ = 102.19 (3)°	0.34 × 0.26 × 0.18 mm
*V* = 857.4 (5) Å^3^	

### Data collection

**Table:**

Rigaku R-AXIS RAPID diffractometer	3009 independent reflections
Radiation source: fine-focus sealed tube	2002 reflections with *I* > 2σ(*I*)
graphite	*R*_int_ = 0.023
ω scans	θ_max_ = 25.0°, θ_min_ = 3.4°
Absorption correction: multi-scan (ABSCOR; Higashi, 1995)	*h* = −10→10
*T*_min_ = 0.964, *T*_max_ = 0.973	*k* = −12→12
6803 measured reflections	*l* = −12→12

### Refinement

**Table:**

Refinement on *F*^2^	Secondary atom site location: difference Fourier map
Least-squares matrix: full	Hydrogen site location: inferred from neighbouring sites
*R*[*F*^2^ > 2σ(*F*^2^)] = 0.035	H-atom parameters constrained
*wR*(*F*^2^) = 0.128	*w* = 1/[σ^2^(*F*_o_^2^) + (0.0299*P*)^2^ + 1.2547*P*] where *P* = (*F*_o_^2^ + 2*F*_c_^2^)/3
*S* = 1.24	(Δ/σ)_max_ < 0.001
3009 reflections	Δρ_max_ = 0.42 e Å^−^^3^
272 parameters	Δρ_min_ = −0.46 e Å^−^^3^
0 restraints	Extinction correction: SHELXL97 (Sheldrick, 2008), Fc^*^=kFc[1+0.001xFc^2^λ^3^/sin(2θ)]^-1/4^
Primary atom site location: structure-invariant direct methods	Extinction coefficient: 0.012 (2)

### Special details

**Table:**

Geometry. All e.s.d.'s (except the e.s.d. in the dihedral angle between two l.s. planes) are estimated using the full covariance matrix. The cell e.s.d.'s are taken into account individually in the estimation of e.s.d.'s in distances, angles and torsion angles; correlations between e.s.d.'s in cell parameters are only used when they are defined by crystal symmetry. An approximate (isotropic) treatment of cell e.s.d.'s is used for estimating e.s.d.'s involving l.s. planes.
Refinement. Refinement of *F*^2^ against ALL reflections. The weighted *R*-factor *wR* and goodness of fit *S* are based on *F*^2^, conventional *R*-factors *R* are based on *F*, with *F* set to zero for negative *F*^2^. The threshold expression of *F*^2^ > σ(*F*^2^) is used only for calculating *R*-factors(gt) *etc*. and is not relevant to the choice of reflections for refinement. *R*-factors based on *F*^2^ are statistically about twice as large as those based on *F*, and *R*- factors based on ALL data will be even larger.

### Fractional atomic coordinates and isotropic or equivalent isotropic displacement parameters (Å^2^)

**Table:**

	*x*	*y*	*z*	*U*_iso_*/*U*_eq_	
B1	0.6460 (5)	0.4668 (4)	0.7888 (4)	0.0240 (8)	
B2	0.7413 (4)	0.3345 (4)	0.6060 (4)	0.0222 (8)	
B3	0.5338 (4)	0.1936 (4)	0.6602 (4)	0.0247 (8)	
B4	0.6535 (4)	0.6248 (4)	1.0187 (4)	0.0209 (7)	
B5	0.4617 (5)	0.6104 (4)	0.8040 (4)	0.0233 (8)	
B6	0.7437 (4)	0.3478 (4)	0.3829 (4)	0.0225 (8)	
B7	0.9709 (5)	0.3173 (4)	0.5343 (4)	0.0252 (8)	
O1	0.7649 (3)	0.4597 (2)	0.7304 (2)	0.0238 (5)	
O2	0.6220 (3)	0.1961 (2)	0.5811 (2)	0.0279 (5)	
O3	0.5410 (3)	0.3195 (2)	0.7601 (2)	0.0266 (5)	
O4	0.7324 (3)	0.5555 (2)	0.9474 (2)	0.0231 (5)	
O5	0.5160 (3)	0.6519 (2)	0.9489 (2)	0.0259 (5)	
O6	0.5330 (3)	0.5377 (2)	0.7283 (2)	0.0248 (5)	
O7	0.6718 (3)	0.3576 (2)	0.4736 (2)	0.0263 (5)	
O8	0.8946 (3)	0.3301 (3)	0.4095 (2)	0.0297 (6)	
O9	0.9009 (3)	0.3166 (2)	0.6244 (2)	0.0260 (5)	
O10	0.4275 (3)	0.0652 (3)	0.6413 (3)	0.0447 (7)	
H10A	0.4225	−0.0042	0.5733	0.067*	
O11	0.7074 (3)	0.6690 (2)	1.1630 (2)	0.0269 (5)	
H11A	0.6297	0.6722	1.1852	0.040*	
O12	0.3292 (3)	0.6490 (3)	0.7475 (2)	0.0284 (5)	
O13	1.1239 (3)	0.3064 (3)	0.5617 (2)	0.0374 (6)	
H13A	1.1659	0.3147	0.6433	0.056*	
N1	0.3291 (3)	0.3057 (3)	0.4112 (3)	0.0329 (7)	
H1A	0.4353	0.3287	0.4654	0.049*	
H1B	0.2747	0.3228	0.4662	0.049*	
H1C	0.3203	0.3605	0.3633	0.049*	
N2	−0.1153 (3)	0.4082 (3)	0.0950 (3)	0.0320 (7)	
H2A	−0.1372	0.4667	0.0531	0.048*	
H2B	−0.0078	0.4427	0.1513	0.048*	
H2C	−0.1730	0.4060	0.1471	0.048*	
C1	0.0788 (4)	0.1079 (4)	0.2176 (4)	0.0316 (8)	
C2	0.0378 (4)	0.1765 (4)	0.1291 (4)	0.0313 (8)	
H2D	0.1214	0.2350	0.1185	0.038*	
C3	−0.1245 (4)	0.1600 (4)	0.0564 (4)	0.0310 (8)	
C4	−0.2492 (4)	0.0650 (4)	0.0667 (4)	0.0380 (9)	
H4A	−0.3593	0.0505	0.0165	0.046*	
C5	−0.2107 (5)	−0.0074 (4)	0.1508 (4)	0.0452 (10)	
H5A	−0.2949	−0.0713	0.1563	0.054*	
C6	−0.0475 (5)	0.0143 (4)	0.2272 (4)	0.0387 (9)	
H6A	−0.0220	−0.0337	0.2850	0.046*	
C7	0.2573 (5)	0.1467 (4)	0.3083 (4)	0.0413 (9)	
H7A	0.2681	0.0865	0.3600	0.050*	
H7B	0.3166	0.1274	0.2483	0.050*	
C8	−0.1624 (5)	0.2555 (4)	−0.0168 (4)	0.0373 (9)	
H8A	−0.2794	0.2172	−0.0789	0.045*	
H8B	−0.1006	0.2570	−0.0744	0.045*	

### Atomic displacement parameters (Å^2^)

**Table:**

	*U*^11^	*U*^22^	*U*^33^	*U*^12^	*U*^13^	*U*^23^
B1	0.0293 (19)	0.027 (2)	0.0191 (19)	0.0131 (16)	0.0134 (16)	0.0092 (16)
B2	0.0212 (18)	0.025 (2)	0.0231 (19)	0.0095 (15)	0.0124 (16)	0.0104 (16)
B3	0.0267 (19)	0.024 (2)	0.024 (2)	0.0084 (16)	0.0132 (16)	0.0098 (17)
B4	0.0219 (18)	0.0198 (18)	0.0207 (19)	0.0057 (14)	0.0104 (15)	0.0081 (15)
B5	0.0287 (19)	0.0248 (19)	0.0197 (19)	0.0128 (16)	0.0115 (16)	0.0101 (16)
B6	0.0245 (19)	0.0242 (19)	0.0179 (18)	0.0128 (15)	0.0081 (15)	0.0060 (15)
B7	0.0265 (19)	0.032 (2)	0.0162 (18)	0.0142 (17)	0.0085 (16)	0.0072 (16)
O1	0.0236 (11)	0.0247 (12)	0.0230 (12)	0.0071 (9)	0.0145 (10)	0.0067 (10)
O2	0.0325 (12)	0.0254 (12)	0.0273 (13)	0.0089 (10)	0.0197 (11)	0.0073 (10)
O3	0.0327 (13)	0.0219 (12)	0.0295 (13)	0.0083 (10)	0.0211 (11)	0.0095 (10)
O4	0.0253 (11)	0.0272 (12)	0.0160 (11)	0.0119 (9)	0.0086 (9)	0.0071 (10)
O5	0.0291 (12)	0.0354 (13)	0.0188 (12)	0.0186 (10)	0.0117 (10)	0.0119 (10)
O6	0.0292 (12)	0.0320 (13)	0.0190 (11)	0.0182 (10)	0.0119 (10)	0.0113 (10)
O7	0.0251 (12)	0.0394 (14)	0.0243 (12)	0.0182 (10)	0.0145 (10)	0.0168 (11)
O8	0.0280 (12)	0.0458 (15)	0.0253 (13)	0.0196 (11)	0.0161 (10)	0.0183 (11)
O9	0.0248 (12)	0.0392 (14)	0.0237 (12)	0.0175 (10)	0.0153 (10)	0.0157 (11)
O10	0.0624 (17)	0.0251 (14)	0.0473 (16)	0.0051 (12)	0.0399 (15)	0.0069 (12)
O11	0.0280 (12)	0.0377 (14)	0.0196 (12)	0.0158 (10)	0.0130 (10)	0.0123 (10)
O12	0.0315 (13)	0.0418 (14)	0.0240 (12)	0.0234 (11)	0.0155 (10)	0.0178 (11)
O13	0.0271 (13)	0.0688 (18)	0.0272 (13)	0.0275 (13)	0.0146 (11)	0.0238 (13)
N1	0.0276 (15)	0.0430 (18)	0.0291 (16)	0.0123 (13)	0.0137 (13)	0.0152 (14)
N2	0.0338 (16)	0.0351 (17)	0.0316 (16)	0.0160 (13)	0.0139 (13)	0.0170 (14)
C1	0.0338 (19)	0.0238 (18)	0.0276 (19)	0.0091 (15)	0.0082 (15)	0.0054 (15)
C2	0.0323 (19)	0.0286 (19)	0.0274 (19)	0.0091 (15)	0.0107 (16)	0.0085 (15)
C3	0.0325 (19)	0.0279 (19)	0.0270 (19)	0.0117 (15)	0.0102 (16)	0.0072 (15)
C4	0.0290 (19)	0.029 (2)	0.045 (2)	0.0075 (16)	0.0120 (17)	0.0087 (18)
C5	0.044 (2)	0.033 (2)	0.054 (3)	0.0065 (18)	0.024 (2)	0.014 (2)
C6	0.050 (2)	0.028 (2)	0.043 (2)	0.0132 (17)	0.0213 (19)	0.0194 (18)
C7	0.041 (2)	0.037 (2)	0.038 (2)	0.0188 (18)	0.0109 (18)	0.0096 (18)
C8	0.041 (2)	0.038 (2)	0.0250 (19)	0.0164 (17)	0.0063 (16)	0.0113 (17)

### Geometric parameters (Å, °)

**Table:**

B1—O1	1.421 (4)	O13—H13A	0.8200
B1—O3	1.480 (4)	N1—C7	1.489 (5)
B1—O4	1.496 (4)	N1—H1A	0.8900
B1—O6	1.499 (4)	N1—H1B	0.8900
B2—O1	1.442 (4)	N1—H1C	0.8900
B2—O9	1.463 (4)	N2—C8	1.497 (4)
B2—O2	1.477 (4)	N2—H2A	0.8900
B2—O7	1.509 (4)	N2—H2B	0.8900
B3—O2	1.354 (4)	N2—H2C	0.8900
B3—O3	1.358 (4)	C1—C2	1.385 (5)
B3—O10	1.370 (4)	C1—C6	1.391 (5)
B4—O4	1.351 (4)	C1—C7	1.495 (5)
B4—O11	1.370 (4)	C2—C3	1.383 (5)
B4—O5	1.386 (4)	C2—H2D	0.9300
B5—O6	1.338 (4)	C3—C4	1.394 (5)
B5—O12	1.377 (4)	C3—C8	1.492 (5)
B5—O5	1.380 (4)	C4—C5	1.377 (6)
B6—O7	1.338 (4)	C4—H4A	0.9300
B6—O8	1.377 (4)	C5—C6	1.383 (5)
B6—O12^i^	1.388 (4)	C5—H5A	0.9300
B7—O9	1.332 (4)	C6—H6A	0.9300
B7—O13	1.368 (4)	C7—H7A	0.9700
B7—O8	1.388 (4)	C7—H7B	0.9700
O10—H10A	0.8200	C8—H8A	0.9700
O11—H11A	0.8200	C8—H8B	0.9700
O12—B6^i^	1.388 (4)		
			
O1—B1—O3	112.6 (3)	C7—N1—H1B	109.5
O1—B1—O4	109.6 (3)	H1A—N1—H1B	109.5
O3—B1—O4	107.5 (2)	C7—N1—H1C	109.5
O1—B1—O6	111.1 (3)	H1A—N1—H1C	109.5
O3—B1—O6	107.2 (3)	H1B—N1—H1C	109.5
O4—B1—O6	108.6 (2)	C8—N2—H2A	109.5
O1—B2—O9	108.6 (3)	C8—N2—H2B	109.5
O1—B2—O2	112.9 (2)	H2A—N2—H2B	109.5
O9—B2—O2	109.0 (3)	C8—N2—H2C	109.5
O1—B2—O7	110.2 (3)	H2A—N2—H2C	109.5
O9—B2—O7	110.1 (2)	H2B—N2—H2C	109.5
O2—B2—O7	105.9 (3)	C2—C1—C6	118.9 (3)
O2—B3—O3	121.8 (3)	C2—C1—C7	118.6 (3)
O2—B3—O10	122.5 (3)	C6—C1—C7	122.2 (3)
O3—B3—O10	115.7 (3)	C3—C2—C1	121.5 (3)
O4—B4—O11	120.4 (3)	C3—C2—H2D	119.2
O4—B4—O5	121.5 (3)	C1—C2—H2D	119.2
O11—B4—O5	118.1 (3)	C2—C3—C4	118.6 (3)
O6—B5—O12	124.5 (3)	C2—C3—C8	120.2 (3)
O6—B5—O5	121.6 (3)	C4—C3—C8	120.9 (3)
O12—B5—O5	113.8 (3)	C5—C4—C3	120.5 (3)
O7—B6—O8	122.9 (3)	C5—C4—H4A	119.8
O7—B6—O12^i^	122.9 (3)	C3—C4—H4A	119.8
O8—B6—O12^i^	114.2 (3)	C4—C5—C6	120.3 (4)
O9—B7—O13	120.9 (3)	C4—C5—H5A	119.8
O9—B7—O8	122.5 (3)	C6—C5—H5A	119.8
O13—B7—O8	116.6 (3)	C5—C6—C1	120.1 (3)
B1—O1—B2	123.5 (3)	C5—C6—H6A	120.0
B3—O2—B2	122.1 (3)	C1—C6—H6A	120.0
B3—O3—B1	121.8 (2)	N1—C7—C1	109.5 (3)
B4—O4—B1	119.7 (3)	N1—C7—H7A	109.8
B5—O5—B4	118.9 (2)	C1—C7—H7A	109.8
B5—O6—B1	120.7 (2)	N1—C7—H7B	109.8
B6—O7—B2	122.4 (2)	C1—C7—H7B	109.8
B6—O8—B7	117.6 (3)	H7A—C7—H7B	108.2
B7—O9—B2	124.2 (3)	C3—C8—N2	108.3 (3)
B3—O10—H10A	109.5	C3—C8—H8A	110.0
B4—O11—H11A	109.5	N2—C8—H8A	110.0
B5—O12—B6^i^	132.5 (3)	C3—C8—H8B	110.0
B7—O13—H13A	109.5	N2—C8—H8B	110.0
C7—N1—H1A	109.5	H8A—C8—H8B	108.4

Symmetry codes: (i) −*x*+1, −*y*+1, −*z*+1.

### Hydrogen-bond geometry (Å, °)

**Table:**

*D*—H···*A*	*D*—H	H···*A*	*D*···*A*	*D*—H···*A*
O10—H10A···O2^ii^	0.82	1.98	2.784 (2)	168
O11—H11A···O3^iii^	0.82	1.84	2.659 (2)	179
O13—H13A···O11^iv^	0.82	2.00	2.815 (2)	173
N1—H1A···O7	0.89	2.08	2.863 (2)	146
N1—H1B···O13^v^	0.89	1.98	2.850 (2)	166
N1—H1C···O6^i^	0.89	2.20	2.916 (2)	137
N1—H1C···O1^i^	0.89	2.54	3.394 (2)	161
N2—H2A···O4^vi^	0.89	1.97	2.822 (2)	159
N2—H2B···O1^i^	0.89	1.99	2.877 (2)	173
N2—H2B···O9^i^	0.89	2.55	3.052 (2)	116
N2—H2C···O12^vii^	0.89	2.19	3.067 (2)	168

Symmetry codes: (ii) −*x*+1, −*y*, −*z*+1; (iii) −*x*+1, −*y*+1, −*z*+2; (iv) −*x*+2, −*y*+1, −*z*+2;  (v) *x*−1, *y*, *z*; (i) −*x*+1, −*y*+1, −*z*+1; (vi) *x*−1, *y*, *z*−1; (vii) −*x*, −*y*+1, −*z*+1.
